# Monoclonal antibody neutralizes *Staphylococcus aureus* serine protease-like protein B (SplB)-induced pathology

**DOI:** 10.1128/iai.00171-25

**Published:** 2025-06-13

**Authors:** Jawad Iqbal, Jessica von Fournier, Nico Wittmann, Murthy N. Darisipudi, Daniel M. Mrochen, Bojan Smiljanov, Kristin Surmann, Gina Wockenfuß, Leif Steil, Thomas P. Kohler, Felix L. Glinka, Shruthi Peringathara, Christopher Saade, Liliane M. Fernandes Hartzig, Uwe Bornscheuer, Christoph A. Reichel, Barbara M. Bröker, Dina Raafat, Silva Holtfreter

**Affiliations:** 1 Institute of Immunology, University Medicine Greifswald 60634, Greifswald, Germany; 2 Pediatric Rheumatology, Department of Pediatric and Adolescent Medicine, University Medicine Greifswald 60634, Greifswald, Germany; 3 Department of Otorhinolaryngology, University Hospital, Ludwig-Maximilians-University Munich, Munich, Germany; 4 Walter Brendel Centre of Experimental Medicine, University Hospital, Ludwig-Maximilians-University Munich, Munich, Germany; 5 Department of Functional Genomics, Interfaculty Institute for Genetics and Functional Genomics, University Medicine Greifswald 60634, Greifswald, Germany; 6 Department of Molecular Genetics and Infection Biology, Interfaculty Institute for Genetics and Functional Genomics, Center for Functional Genomics of Microbes, University of Greifswald 26552, Greifswald, Germany; 7 Department of Biotechnology and Enzyme Catalysis, Institute of Biochemistry, University of Greifswald 26552, Greifswald, Germany; 8 Comprehensive Cancer Center (CCC) Munich Ludwig-Maximilians-University (LMU), LMU Hospital 27192 , Munich, Germany; 9 Department of Microbiology and Immunology, Faculty of Pharmacy, Alexandria University 540354, Alexandria, Egypt; St Jude Children's Research Hospital, Memphis, Tennessee, USA

**Keywords:** bacterial protease, host-pathogen interaction, vaccine candidate, passive vaccination, neutralization, antibody characterization

## Abstract

*Staphylococcus aureus* is a versatile pathogen, renowned for its arsenal of virulence and immune evasion factors. Several *S. aureus* virulence factors have been targeted in vaccination trials; however, so far, without success. Promising new vaccine candidates are the staphylococcal serine protease-like proteins (Spl A–F), which are involved in the pathogenesis and immune evasion of *S. aureus*. SplB, for instance, promotes type 2 immune responses and inactivates human complement factors. In this study, we report on the production and characterization of a murine monoclonal antibody (mAb) against SplB. The murine anti-SplB mAb α-SplB1 was produced by hybridoma technology, and its binding characteristics were investigated using enzyme-linked immunosorbent assay (ELISA), Western blot, and MicroScale Thermophoresis. Its neutralizing capacity was determined in a fluorogenic substrate assay, Western blot, and a murine vascular leakage model. α-SplB1 bound to recombinant SplB with high specificity, showing no cross-reactivity to other Spls or secreted proteins of *S. aureus*. MicroScale Thermophoresis revealed a K_D_ value of 37.9 nM for the α-SplB1:SplB interaction. α-SplB1 neutralized the enzymatic activity of SplB *in vitro* in a dose-dependent manner, yielding complete neutralization at a twofold molar excess of the antibody. In a murine vascular leakage model, the antibody completely abolished SplB-mediated endothelial damage. In summary, we produced a neutralizing mAb against the staphylococcal protease SplB, which merits further investigation as a candidate for the immunotherapy of SplB-induced pathologies.

## INTRODUCTION

*Staphylococcus aureus* is a Gram-positive opportunistic human pathogen and a leading cause of various infections ranging from mild to severe, such as skin and soft tissue infections, infective endocarditis, sepsis, and necrotizing pneumonia (1, 2). These bacteria account for 9.0% of nosocomial infections and 15.4% of hospital-acquired sepsis cases in Europe (3). The treatment of staphylococcal infections is often hampered by multiple antibiotic resistances (4). Thus, new prophylactic and therapeutic approaches are urgently needed, including passive and active vaccination strategies (4, 5).

Over the past decades, several clinical trials involving active or passive vaccines targeting *S. aureus* proteins have failed (6). A major obstacle in vaccine development is the broad and highly redundant array of staphylococcal virulence factors (6–8), which can facilitate barrier disruption, cause host tissue damage, and contribute to the persistence and spread of infections (1). Moreover, numerous staphylococcal immune evasion molecules block or manipulate innate and adaptive immune responses (8).

Over the past years, staphylococcal serine protease-like proteins (Spls) have emerged as important virulence and immune evasion factors. These secreted proteases are encoded on the νSaβ genomic island in an operon comprising up to six genes: *splA*, *splB*, *splC*, *splD*, *splE*, and *splF* (9, 10). The composition of the *spl* operon is diverse and closely linked to the clonal background of the *S. aureus* isolates (11–13). SplB is found in about 60% of *S. aureus* isolates from healthy carriers (11, 12). The *spl* operon is regulated alongside other *S. aureus* virulence factors, suggesting its involvement in virulence (14). Furthermore, an *S. aureus* mutant expressing only the *spl* operon displayed hypervirulence in a murine sepsis model compared with a strain lacking all secreted proteases (15). Interestingly, the Spls favor a type 2 immune response, characterized by anti-Spl-IgE and -IgG4 antibodies as well as a Spl-specific Th2 memory response in healthy individuals (16). Intratracheal administration of SplD also causes an asthma-like phenotype in mice (16, 17). Besides their role as allergens, Spls also interfere with other immune defense mechanisms. For instance, SplB inactivates central human complement proteins, thereby contributing to *S. aureus* immune evasion (11). Given its multifaceted involvement in staphylococcal pathogenesis, SplB represents an interesting target for both active and passive vaccination strategies.

Several studies have investigated the suitability of SplB as a vaccine candidate. This protease possesses high immunogenicity in mice. For instance, an intraperitoneal (i.p.) vaccination of mice with non-adjuvanted SplB elicits robust antibody levels and a Th2-dominated T-cell response, which further underscores the type 2 immune polarizing capacity of this protease (18). Vaccination with SplB, alongside the pore-forming toxins LukE and LukS-PV, and the cysteine protease SspB, provided modest protection against *S. aureus*-induced dermonecrosis in C57BL/6 mice (19). Antibody levels against SplB in the general human population show a high degree of variability with a range spanning 3.5 logs (20). This high variability might be attributed to the history of exposure and suggests that a considerable proportion of individuals lack protective antibody levels.

The aim of this work was to generate a monoclonal antibody (mAb) against SplB, which is capable of neutralizing SplB-mediated pathologies. Using hybridoma technology, we generated a murine mAb (α-SplB1) that specifically bound to SplB, showing no cross-reactivity to other Spl proteases, and neutralized its enzymatic activity *in vitro*. Moreover, α-SplB1 effectively neutralized SplB-mediated vascular leakage in a mouse model. Taken together, these promising findings encourage further investigations of α-SplB1 as a candidate for a passive vaccine against SplB-related diseases.

## RESULTS

### α-SplB1 binds to recombinant SplB *in vitro*

Using the hybridoma technology, we generated a murine mAb (α-SplB1) against the *S. aureus* protease SplB. Isotyping revealed that α-SplB1 belongs to the IgG1 subclass and has κ light chains (data not shown). The DNA sequences of the Fab region of the heavy and light chains are provided in Fig. S1. The coding variable regions of the heavy and light chains of α-SplB1 showed 94.9% and 98.6% sequence similarity to the germline sequence, respectively (Fig. S2).

**Fig 1:**
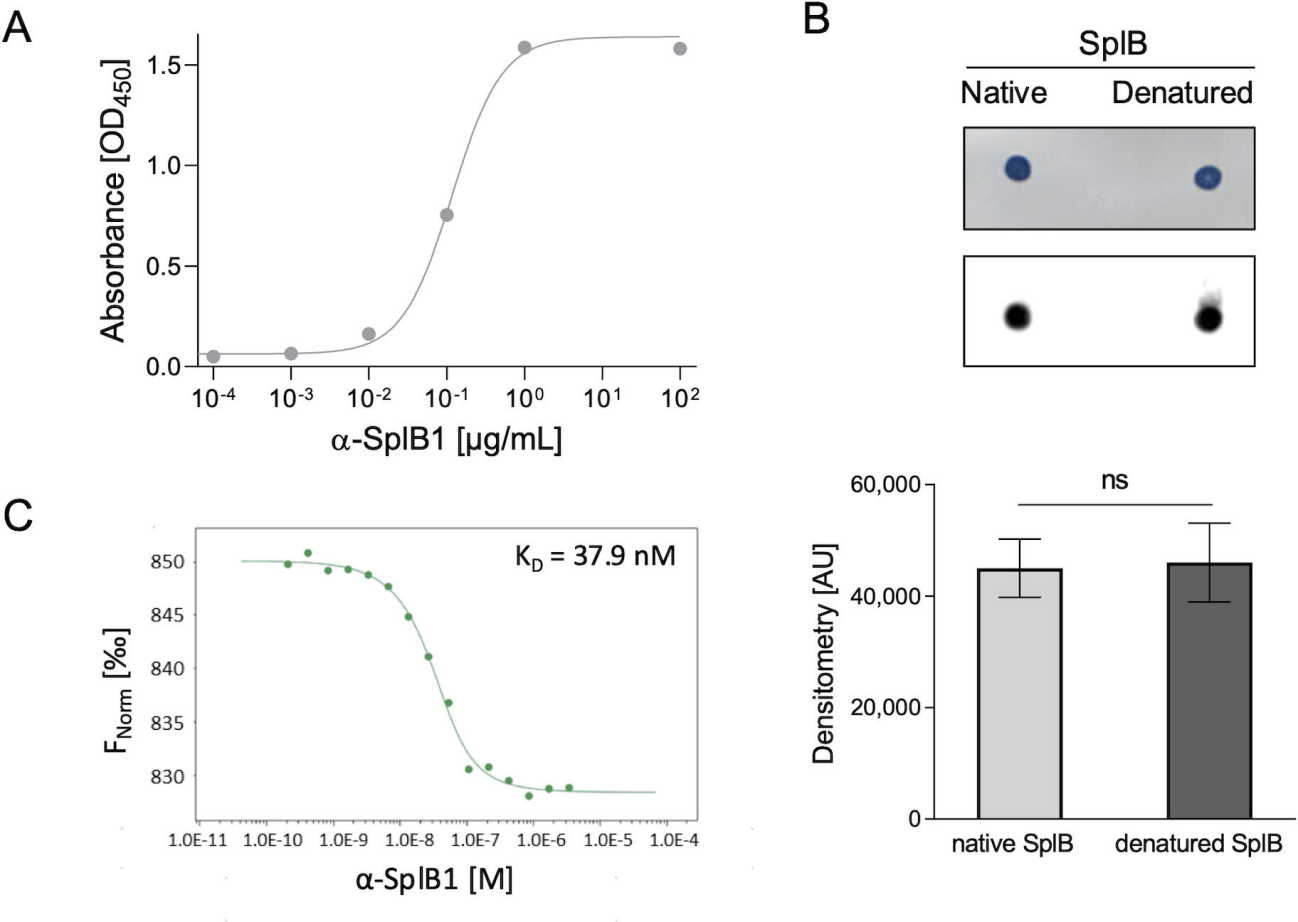
α-SplB1 mAb binds SplB in a concentration-dependent manner. (**A**) Binding of purified α-SplB1 mAb to recombinant tag-free SplB was determined by ELISA. One of three similar experiments is shown. (**B**) Dot blot immunoassay with 1 µg of native or heat-denatured tag-free SplB (upper picture; protein stained with amido black dye) incubated with 10 ng/mL α-SplB1. The graph (lower picture) shows the signal intensities of the dot blot spots as determined using ImageJ 1.52a, presented as mean ± SD of three independent experiments. Statistics: Unpaired *t*-test, ns, not significant. (**C**) Binding affinity of α-SplB1 to tag-free SplB was determined by MicroScale Thermophoresis and is depicted as normalized fluorescence (F_Norm_ [‰]). α-SplB1 was titrated to a constant amount of labeled, tag-free SplB. One out of three similar experiments is shown. The depicted K_D_ is the mean of three replicates.

In an indirect enzyme-linked immunosorbent assay (ELISA), α-SplB1 bound to the tag-free SplB protein with a half-maximal effective concentration (EC_50_) of 0.12 µg/mL (Fig. 1A). α-SplB1 bound to both native and denatured SplB in a similar manner (Fig. 1B)**,** suggesting that it recognizes a linear, surface-exposed epitope. The binding affinity of α-SplB1 to the tag-free SplB was determined using MicroScale Thermophoresis (MST). α-SplB1 (3.42–0.000209 µM) showed a concentration-dependent binding to its target, with a calculated dissociation constant (K_D_) of 37.9 nM (Fig. 1C). The thermal stability of the SplB antibody was determined with an onset of 58 ± 0.07°C and a melting temperature of 69.1 ± 0.05°C (Fig. S3).

### α-SplB1 does not cross-react with other *S. aureus* Spls

**Fig 2:**
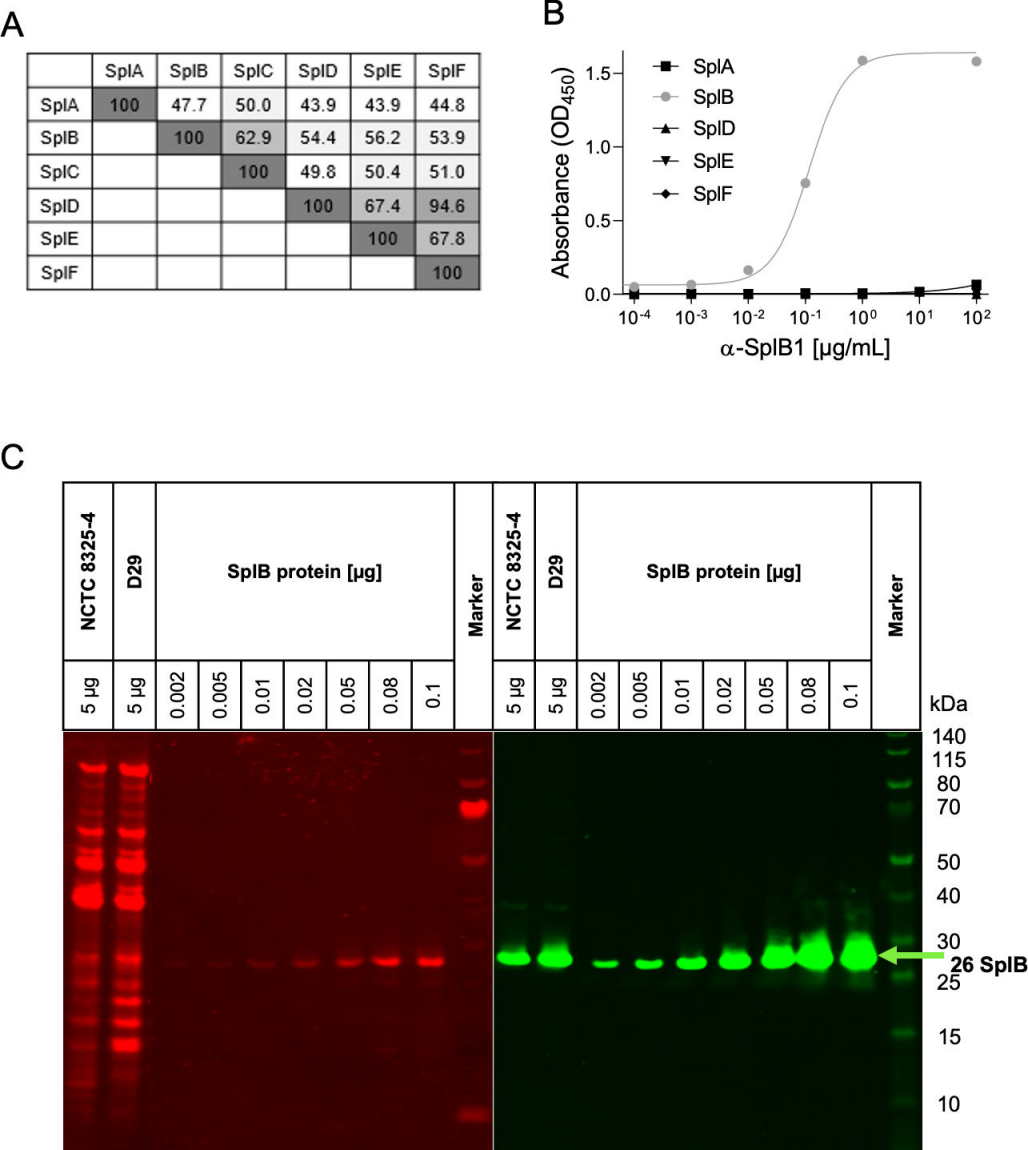
α-SplB1 mAb does not cross-react with other *S. aureus* proteins. (**A**) Amino acid sequence identities among the Spl proteins based on paired alignments. Sequences were derived from *S. aureus* NCTC 8325-4. (**B**) A potential cross-reactivity of α-SplB1 with other Spl proteases was evaluated by ELISA using recombinant tag-free SplA, SplD, SplE, and SplF. (**C**) Extracellular proteins (ECPs) from TSB culture supernatants of *S. aureus* NCTC 8325-4 and D29 were harvested in the stationary growth phase. Quantitative Western blot was performed with 5 µg of ECPs and purified tag-free SplB in different amounts (0.002–0.1 µg). The left panel shows total protein staining using Revert 700 total protein stain (LI-COR Biosciences, Lincoln, USA [red]); the right panel shows the Western blot signals using murine α-SplB1 (300 ng/mL) as primary antibody and an IRDye 800CW-labeled goat anti-mouse IgG Ab (1:10,000 dilution) as secondary antibody (green). Prestained PageRuler (ThermoFisher Scientific, Waltham, USA) served as a size marker.

SplB shows 47.7%–62.9% amino acid sequence homology to the other Spl proteins (Fig. 2A). We therefore studied whether α-SplB1 cross-reacts with other recombinant Spl proteins (SplA, SplD, SplE, and SplF) using an indirect ELISA. α-SplB1 showed no affinity towards SplA, SplD, SplE, or SplF (Fig. 2B).

To further investigate the specificity of α-SplB1 for SplB, we tested if the antibody recognizes native SplB in the staphylococcal secretome by using a Western blot. A total of 5 µg of extracellular proteins (ECPs) of *S. aureus* strains NCTC 8325-4 (laboratory isolate, ST8, MSSA) (21) and D29 (Danish USA300 isolate, ST8, CA-MRSA) (22) obtained from the stationary phase of a TSB culture were separated by SDS-PAGE, blotted, and incubated with α-SplB1. As a control, we performed the Western blot with increasing amounts (0.002–0.1 µg) of recombinant SplB. α-SplB1 specifically bound to SplB in the ECP preparation of both *S. aureus* strains and did not cross-react with other abundant staphylococcal proteins (Fig. 2C). The absolute amount of SplB in the ECP preparation was calculated using an SplB dilution series (Fig. S4). The ECPs of *S. aureus* NCTC 8325-4 and D29 contained 2.8 and 6.0 ng SplB per 1 µg total protein, respectively. The concentration of SplB in the supernatants of *S. aureus* strains NCTC 8325-4 and D29 during stationary growth phase in TSB was determined at 527 and 541 ng/mL, respectively (Fig. S4). Taken together, these results indicate that α-SplB1 is highly specific for SplB and does not cross-react with the other Spl proteases or other secreted proteins of *S. aureus*.

### α-SplB1 neutralizes the enzymatic activity of SplB

**Fig 3:**
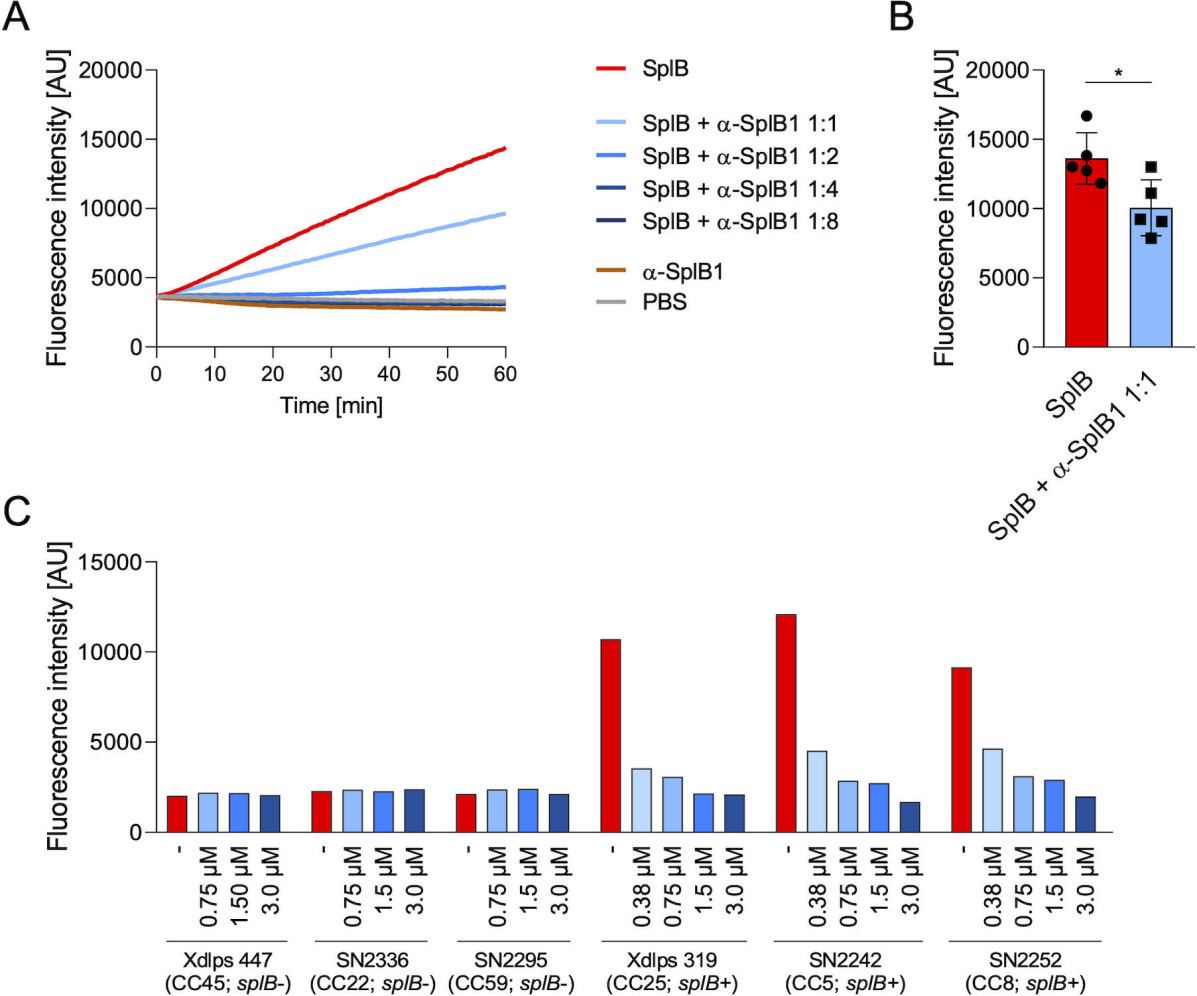
α-SplB1 mAb efficiently neutralizes the enzymatic activity of SplB *in vitro*. (**A**) The neutralizing activity of α-SplB1 mAb was assessed using the synthetic peptide substrate Ac-VEID-MCA. Then, 2.5 µM tag-free SplB was pre-incubated with α-SplB1 in triplicates at the indicated molar ratios at 37°C for 1 h. After substrate addition, fluorescence was quantified over 60 min. A total of 2.5 µM SplB alone and 5 µM α-SplB1 mAb alone served as negative controls. One out of five similar experiments is depicted. (**B**) Comparison of fluorescence intensity at t = 60 min between SplB and SplB + αSplB1 at a 1:1 molar ratio. Mean ± SD is shown. Statistics: Unpaired *t*-test, *P < 0.05. (**C**) Culture supernatants (TSB, stationary phase) from three *splB*-negative and three *splB*-positive *S. aureus* isolates were concentrated 250-fold to enrich the secreted SplB, incubated with the α-SplB1 mAb for 1 h at varying concentrations or PBS as control, and subsequently screened for AMC substrate cleavage. One out of two experiments is shown. AU, arbitrary units.

To test whether α-SplB1 can neutralize the enzymatic activity of the serine protease SplB, we developed an *in vitro* assay using the synthetic SplB substrate Ac-VEID-MCA. α-SplB1 was able to neutralize SplB in a dose-dependent manner. The enzymatic activity of SplB was neutralized by 66% when α-SplB1 was used at a 1:1 molar ratio and completely abolished at a 1:2 molar ratio (SplB:α-SplB1) (Fig. 3A and B). To investigate whether the α-SplB1 mAb can also neutralize the natural SplB protein secreted by *S. aureus,* we prepared culture supernatants (TSB, stationary phase) from three *splB*-negative *S. aureus* isolates (lineages CC45, CC22, and CC59) and three *splB*-positive isolates (CC25, CC5, and CC8). These supernatants were concentrated 250-fold to enrich the secreted SplB, incubated with the α-SplB1 mAb for 1 h, and subsequently screened for substrate cleavage (Fig. 3C). Substrate cleavage was observed only for supernatants from *splB*-coding strains. Again, the enzymatic activity of the natural SplB enzyme was completely neutralized in all *splB*-positive bacterial supernatants.

We also tested whether α-SplB1 inhibits the cleavage of a natural SplB substrate: human complement factor C3 (11). C3 cleavage products were visualized by Western blotting. Indeed, α-SplB1 completely blocked the SplB-induced cleavage of the alpha chain of the human complement factor C3 at a 1:2 molar ratio (Fig. S5). Overall, these data demonstrate that α-SplB1 effectively neutralizes the enzymatic activity of SplB *in vitro*.

### α-SplB1 blocks SplB-induced vascular leakage

**Fig 4:**
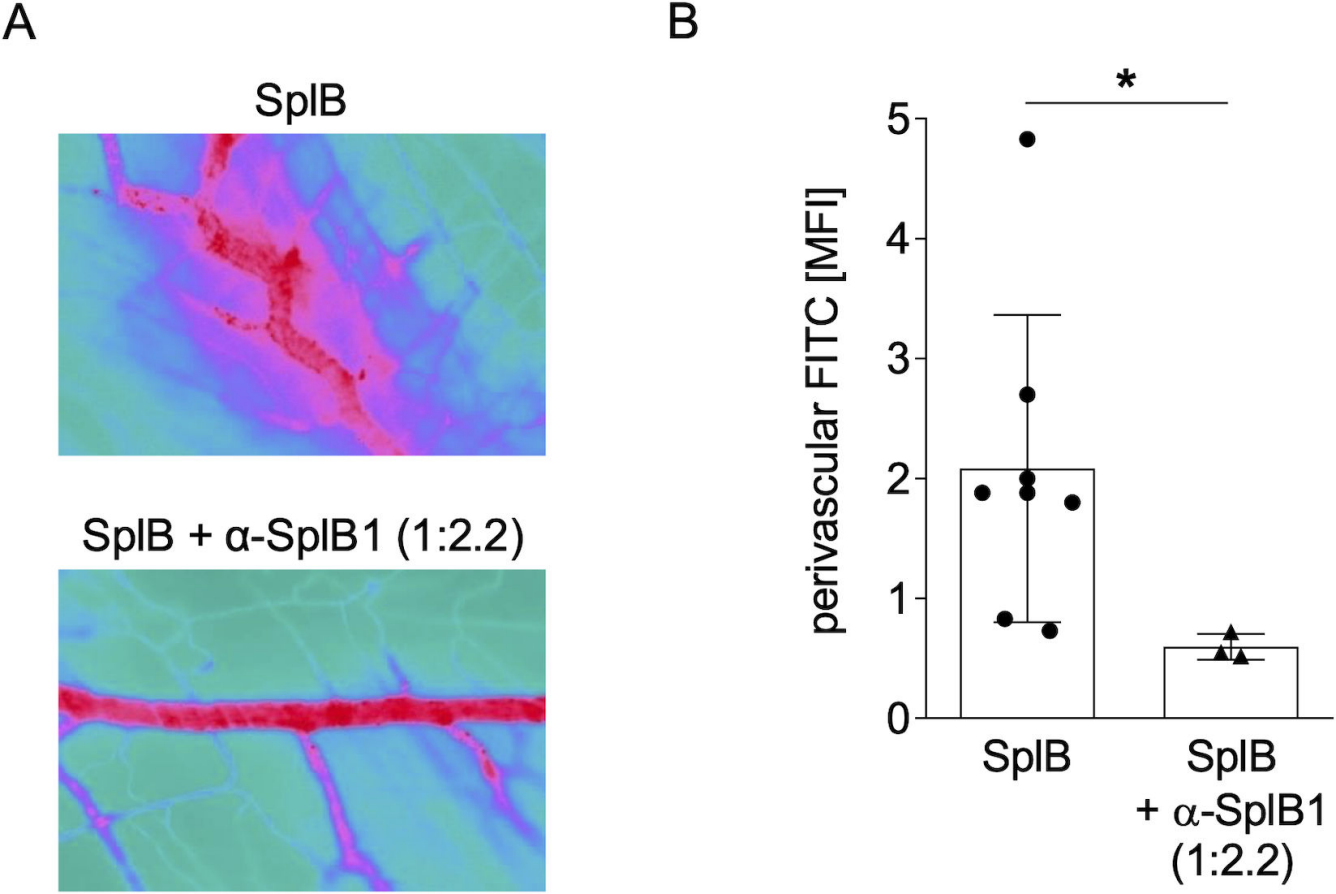
α-SplB1 mAb blocks SplB-induced vascular leakage in a murine cremaster muscle microvascular leakage model. (**A**) Male 6–8-week-old C57BL/6N mice were intrascrotally challenged with 10 µg tag-free SplB, with or without prior administration of α-SplB1 mAb. Vascular injury was assessed by examining the leakage of intravenously administered FITC-dextran into the perivascular tissue through fluorescence microscopy. FITC-dextran leakage was captured using a CCD camera and is presented in false colors. SplB induced vascular leakage, as indicated by a perivascular spread of the FITC signals (upper panel). Pretreatment with α-SplB1 mAb prevented SplB-induced vascular leakage (lower panel). (**B**) Quantification of the perivascular FITC signal using ImageJ software (mean MFI ± SD) from 3 to 8 mice per experimental group. Statistics: Unpaired *t*-test, *P < 0.05.

*S. aureus* secretes several virulence factors, including hemolysins and proteases, that interact with epithelial and endothelial receptors, enabling barrier disruption and tissue invasion (23–27). Based on these data, we hypothesized that SplB can disrupt the endothelial barrier. To test our hypothesis, we used a cremaster mouse model, which is commonly used to visualize microcirculation and vascular injury in mice by intravital microscopy (28). Administration of 10 µg of SplB led to pronounced leakage of FITC-dextran into the perivascular space in the murine cremaster muscle, indicating that SplB is able to cause endothelial damage (Fig. 4). Treatment of mice with 150 µg of α-SplB1 1 h prior to SplB administration reduced the leakage of FITC-dextran significantly, demonstrating that α-SplB1 blocks the enzymatic activity of SplB *in vivo* if applied in a 1:2.2 molar ratio.

## DISCUSSION

Recent work from our research group has identified SplB as a promising candidate for passive vaccination. SplB has allergenic properties both in humans and mice (16, 17, 29). Moreover, it cleaves central components of the complement system, thereby inhibiting all complement activation pathways and inhibiting opsonophagocytosis by neutrophils (11). Here, we isolated and characterized a mAb (α-SplB1) that specifically targets SplB, neutralizes its enzymatic activity *in vitro,* and completely abolishes SplB-induced vascular damage *in vivo*.

α-SplB1 displayed average binding affinity towards SplB in an MST assay, with a K_D_ value of 37.9 nM. The K_D_ value for antigen-antibody interactions is frequently in the low micromolar (10^−6^) or nanomolar range (10^−7^ to 10^−9^), while high-affinity antibodies usually have a K_D_ in the low nanomolar range (30). However, the determined K_D_ value is strongly influenced by the MST protocol used. MST relies on changes in thermophoretic mobility induced by ligand-protein interactions and is hence influenced by variables such as buffer composition and pH (31, 32). Therefore, a comparison of the K_D_ values presented in the various publications is inherently challenging.

The DNA sequence of the α-SplB1 mAb showed high similarity to the germline sequence (94.9% and 98.6% for the heavy and light chains, respectively). Upon repeated antigen exposure, antigen-specific B cells undergo affinity maturation, followed by a stringent selection of those B cells that produce antibodies of higher affinity during germinal center reactions (33). Previous studies have shown that the variable regions of such affinity-matured antibodies show a sequence similarity between 45% and 98% compared with the germline sequence (34–37). Nevertheless, germline-like mAbs can also have high affinity and potent neutralizing capacity *in vitro* and in animal models (38). These antibodies can retain a degree of structural plasticity, which allows them to bind to several different antigens (39). However, we could demonstrate that α-SplB1 binds specifically to SplB, but neither to other sequence-related Spls nor to unrelated virulence factors in the staphylococcal secretome, highlighting its high specificity.

Vascular leakage is commonly caused by endothelial barrier disruption due to cell death or disturbance of cellular tight junctions (40). *S. aureus* causes endothelial damage by secreting the pore-forming toxin alpha-hemolysin, which triggers inflammasome activation and pyroptosis (41). Moreover, the *S. aureus* cysteine proteases staphopain A and B induce vascular leakage in the guinea pig model via the activation of the plasma kallikrein/kinin system and the release of bradykinin (27). Using a cremaster mouse model, we demonstrated that SplB disrupts the endothelial barrier *in vivo*, thereby inducing vascular leakage. This may explain why an *S. aureus* USA300 strain containing only the *spl* operon is more virulent in a bloodstream infection model than the one lacking all secreted proteases (15). The endothelial damage mediated by SplB may also contribute to the pathology of several other diseases, including sepsis, endocarditis, and allergy by facilitating *S. aureus* dissemination.

A preventative application of α-SplB1 almost abolished SplB-induced vascular leakage in mice. As α-SplB1 was also shown to abolish SplB enzymatic activity at a 1:2 molar ratio *in vitro*, we conclude that enzymatic activity is required for endothelial leakage. Hence, α-SplB1 could have therapeutic potential by inhibiting *S. aureus* dispersal and preventing leakage-induced shock. However, we did not validate the potential protective role of the α-SplB1 mAb in *S. aureus* infection models, which is a clear limitation of the current study. We refrained from these experiments because we believe that a successful anti-protease approach likely requires the simultaneous targeting of multiple staphylococcal proteases. *S. aureus* secretes up to 10 different proteases, with partially redundant functions (8, 42). For instance, vascular leakage is not only caused by SplB but also by two *S*. *aureus* cysteine proteases, staphopain A (ScpA) and B (SspB) (27). Similarly, both SplB and the *S. aureus* metalloprotease aureolysin mediate complement evasion by proteolytically cleaving complement factors (11, 43). In line with this, *S. aureus* single protease mutants (*aureolysin*, *scpA*, *sspB*) often did not demonstrate attenuated virulence in murine infection models (44, 45). An *spl* operon deletion mutant also revealed no major differences in virulence compared with the parental strain in a rat peritonitis model (10), while the *spl* operon enhanced virulence in a strain that lacked all other secreted proteases in a murine bloodstream infection model (15). Along the same line, a vaccine that aims at preventing *S. aureus*-induced vascular leakage should ideally target not only SplB, but also alpha-hemolysin, staphopain A, and staphopain B. Even though the molecular pathways differ, these other toxins could compensate for the neutralization of SplB in terms of endothelial leakage. Altogether, in the infection context, targeting only SplB might not be sufficient to provide protection from protease-mediated pathologies and/or vascular damage. Testing α-SplB1 in a multi-valent prophylactic or therapeutic approach is beyond the scope of this work but will be addressed in the future.

As bacterial proteases play an important role in tissue invasion, disease pathology, and immune evasion, the development of specific inhibitors might ameliorate the severity of infection and strengthen the host immune defense mechanisms (46). Since there is little structural similarity between prokaryotic and eukaryotic proteases, including their reactive sites, it should be possible to create inhibitors specific to pathogen proteases without affecting host proteases. Compared with small molecules, which frequently induce inhibition by occupying the active site of the enzyme and are highly cell permeable, mAbs have the advantage of outstanding specificity, while also being stable in serum and able to cross the blood-brain barrier (47–50). However, only a few antibodies against bacterial proteases have been characterized so far, and none of them have been approved for therapeutic use (51, 52). This is remarkable since protease inhibitors are used to treat a variety of diseases, ranging from viral infections to thrombosis (53–55).

*S. aureus* is notorious for its pervasive antibiotic resistance, underscoring the necessity for alternative therapeutic approaches. Passive vaccination is a much-discussed strategy to combat infections by several pathogens, but to date, prophylactic antibody treatment has only been approved for *Clostridioides difficile* and respiratory syncytial virus infections in certain at-risk populations (56, 57). Developing vaccines against *S. aureus* has proven to be very challenging. This has been attributed to the multiplicity and redundancy of *S. aureus* virulence factors, as well as the notorious staphylococcal protein A (SpA), which binds the Fc part of IgG (6, 7, 58, 59). To overcome this, active and passive (pre-) clinical vaccine trials are based on multivalent vaccines targeting several virulence factors (59, 60). In addition, pre-clinical studies demonstrate that deleterious SpA effects can be prevented by vaccination against SpA or by engineering human antibodies that avoid sequestration by SpA (61, 62), raising new hopes for an anti-staphylococcal vaccine.

Although it is unlikely that antibody-based therapeutics will replace antibiotics in the near future, they have the potential to significantly enhance the efficacy of antibiotics for the treatment of invasive staphylococcal diseases (7). Conversely, exposure to antibiotics can alter the expression of surface markers, thereby increasing mAb efficiency (63). Hence, it would be interesting to test whether the application of α-SplB1 alongside antibiotics could mitigate disease severity *in vivo*.

Besides their clinical potential, mAbs are an indispensable immunological tool in various research applications. Their strong binding affinity is crucial for various diagnostic assays such as immunohistochemistry, ELISA, Western blots, flow cytometry, as well as for the purification of their target proteins (64, 65). Despite recent advances, the pathomechanisms of SplB are still not fully deciphered. The α-SplB1 mAb will therefore be used to further investigate the complex role of SplB in *S. aureus* colonization and infection.

In conclusion, we generated a neutralizing murine mAb against SplB, which displays a high specificity for its target and can efficiently neutralize the enzymatic activity of SplB both *in vitro* and *in vivo*. Therefore, α-SplB1 is a promising candidate for a multivalent antistaphylococcal vaccine. Passive vaccination could extend the therapeutic portfolio against *S. aureus*, whose virulence and prevalent antibiotic resistance are a major threat to public health.

## MATERIALS AND METHODS

### Expression and purification of recombinant SplB

Recombinant SplB with a C-terminal Strep-tag was cloned from the genome of *S. aureus* NCTC 8325 (SAOUHSC_01941 gene cloned into pPR-IBA1) and overexpressed in *E. coli* BL21(DE3)pLysS in lysogeny broth (LB) medium containing ampicillin (100 µg/mL) and chloramphenicol (25 µg/mL), as described previously (18). Protein expression was induced with isopropyl-β-D-1-thiogalactopyranoside (IPTG; Roth, Karlsruhe, Germany) at a final concentration of 1 mM. About 3–5 h later, cells were harvested by centrifugation (15 min, 4°C, 14,000 × *g*) and lyzed by sonication (30 min, on ice). SplB was then purified from the lysate by affinity chromatography (ÄKTA start, GE Healthcare, Fairfield, USA) using a StrepTrap HP column (GE Healthcare, Fairfield, USA) according to the manufacturer’s instructions. Buffer exchange to 0.5 × phosphate-buffered saline (PBS) was performed using a two-step size exclusion centrifugation with centrifugal filter units (Amicon Ultra 30K/10K, Merck Millipore, Billerica, USA), and endotoxin was removed using the EndoTrap Endotoxin removal system (Hyglos GmbH, Bernried am Starnberger See, Germany).

In order to ensure that the generated mAb is not directed against the Strep-tag, the following ELISA experiments to select promising hybridoma cell clones were conducted with C-terminal His-tagged SplB that was produced and purified as described above, except for the following modifications: (i) the SAOUHSC_01941 gene was cloned into pASK-IBA33+; (ii) anhydrotetracycline was used to induce protein expression; (iii) SplB-His was purified using HisTrap HP columns (GE Healthcare, Fairfield, USA) and eluted with 20 mM sodium phosphate/0.5 M NaCl/500 mM imidazole (pH 7.4) (18).

Tag-free Spls (SplA, SplB, SplD, SplE, and SplF) were recombinantly expressed in *Bacillus subtilis* and purified as previously described (16).

### Generation and purification of anti-SplB mAb (α-SplB1)

The anti-SplB mAb (α-SplB1) was generated by repetitive i.p. immunization of male 8-week-old *S. aureus*-naïve wild-type C57BL/6NRj (C57BL/6N) mice as previously described (18). All animal experiments were approved by the local authorities (LALLF, State Office for Agriculture, Food Safety and Fisheries, Mecklenburg-Western Pomerania, Germany; Az7221.3-2-044/13).

Briefly, mice were immunized on day (d) 0, d21, and d42 with 100, 50, and 30 µg, respectively, of SplB-Strep in 100 µL of physiological NaCl solution supplemented with 100 µL of adjuvant (TiterMax Gold Adjuvant, Sigma-Aldrich, St. Louis, USA). Booster immunization was performed on d63 by retroorbitally injecting the mice with 10 µg antigen (without adjuvant).

Animals were sacrificed on d66, blood was collected, and spleens were aseptically removed and homogenized. Splenocytes were fused with myeloma cells (P3X63Ag8.653) using polyethylene glycol to generate antibody-producing hybridomas. Afterwards, stable clones of hybridoma cells were selected by limiting dilution, and the hybridoma supernatants were tested for SplB-binding using an antigen-specific IgG ELISA with the His-tagged SplB. After selecting promising clones (*n* = 3), the respective hybridoma supernatants were collected for the purification of the murine mAb using Protein-G-Sepharose resin (GE Healthcare, Munich, Germany) following the manufacturer’s instructions. As all three selected clones showed an identical antibody sequence, we continued our study with only one clone (α-SplB1). The concentration of purified α-SplB1 was determined using the mass extinction coefficient of 14.0 at 280 nm for a 1% (w/v) IgG solution.

The isotype of α-SplB1 was determined using a magnetic bead-based kit (Milliplex Map, Mouse Immunoglobulin Isotyping Magnetic Bead Panel, Merck, Billerica, USA) on a multiplex immunoassay system (Bio-Plex 200, Bio-Rad, Hercules, USA) following the manufacturer’s protocol. The data were recorded and analyzed using the Bio-Plex Manager 5.0 software (Bio-Rad, Hercules, USA).

### Sequencing of the variable regions of the heavy and light chains of the purified α-SplB1

To sequence the variable regions of the heavy and light chains of the generated α-SplB1, total RNA was isolated from 1 × 10^6^ hybridoma cells using the RNeasy Kit (Qiagen GmbH, Hilden, Germany) according to the manufacturer’s instructions, and reverse transcribed into cDNA using dNTPs (ThermoFisher Scientific, Waltham, USA) and the Superscript IV first-strand synthesis system (ThermoFisher Scientific, Waltham, USA). The variable regions of the heavy and light chains were amplified by PCR using the primers listed in Table S1 and the Accuprime Taq DNA Polymerase System kit (ThermoFisher Scientific, Waltham, USA) (66). The amplified cDNA samples were separated on a 1% agarose gel. The DNA fragments corresponding to the light and heavy chains (400 bp) were extracted from the gel using the NucleoSpin Gel and PCR Clean Kit (Macherey-Nagel, Düren, Germany) according to the manufacturer’s manual and sent for sequencing (Mix2seq kit, Eurofins Genomics, Ebersberg, Germany). Comparison to the germline sequence and annotations were done using IgBlast (67) (https://www.ncbi.nlm.nih.gov/igblast/) and ImMunoGeneTics (68) (https://www.imgt.org/). Finally, the SnapGene viewer (GSL Biotech, version 5.0.7, SnapGene software [https://www.snapgene.com/]) was used to label the DNA and amino acid sequences of the variable regions of the heavy and light chains (Fig. S2).

### SplB-specific IgG ELISA

96-well microtiter plates (Nunc MaxiSorp, ThermoFisher Scientific, Waltham, USA) were coated with 1 µg/mL of tag-free SplB protein overnight at 4°C. Plates were incubated with either undiluted hybridoma supernatant or serial 10-fold dilutions of purified α-SplB1 (10^2^–10^−4^ µg/mL) for 1 h at room temperature (RT). The plates were then washed, and IgG-binding was detected using a goat anti-mouse IgG conjugated with HRP (1:30,000, Southern Biotech, Birmingham, USA; #1036-05) and BD OptEIA TMB Substrate Reagent Set (BD, Franklin Lakes, USA). The optical density (OD) at 450 nm was measured with the TECAN Infinite M200 plate reader (Tecan Group Ltd., Maennedorf, Switzerland).

### SplB-specific dot blot

A dot blot assay was conducted to visualize the binding of α-SplB1 to native and denatured tag-free SplB. 1 µg of recombinant native or heat-denatured (heated at 95°C for 30 min) SplB was spotted onto a polyvinylidene fluoride membrane (Immobilon-P PVDF Membrane, Merck, Billerica, USA). SplB was stained with amido black dye for 10 min, and the images were taken using a CanoScan LiDE 110 scanner (Canon, Krefeld, Germany). The membrane was then destained with Tris-buffered saline containing 0.05% Tween-20 (TBST) for 5 min and blocked with 5% milk powder in TBST for 1 h at RT. Afterwards, the membrane was incubated overnight at 4°C with 10 ng/mL α-SplB1 to allow the binding of the mAb to its target protein. Unbound antibodies were removed by washing three times with TBST. To visualize the binding of α-SplB1 to its target protein, the membrane was incubated with goat anti–mouse IgG conjugated to POD (1:100,000, Southern Biotech, Birmingham, USA; #1036-05) for 1 h at RT, and then with the substrate (Supersignal West Femto Maximum, ThermoFisher Scientific, Waltham, USA) for 3 min. The signals were detected using a ChemoCam Imager (Intas Science Imaging Instruments GmbH, Göttingen, Germany), and the signal intensities of the spots were quantified using ImageJ 1.52 a (69).

### MicroScale Thermophoresis (MST)

The binding affinity of α-SplB1 to tag-free SplB was assessed using MicroScale Thermophoresis (MST), which quantifies biomolecular interactions in the liquid phase. Briefly, tag-free SplB was labeled using the RED-NHS dye (molar dye:protein ratio 3:1) in the Monolith protein labeling kit RED-NHS 2nd Generation (NanoTemper Technologies GmbH, Munich, Germany) with certain modifications as described elsewhere (70). Samples were diluted in MST buffer supplemented with 0.05% (w/v) Tween-20. The labeled SplB (target, 40 nM) and the α-SplB1 (ligand, serially diluted from 3.42  µM to 0.209  nM) were premixed and loaded into Monolith NT.115 Standard Capillaries. The MST was measured using a Monolith NT.115 instrument (NanoTemper Technologies GmbH, Munich, Germany) at RT using MO Control software 1.6 (NanoTemper Technologies GmbH, Munich, Germany). Instrument parameters were adjusted to 100% excitation power and 40% MST power, and the data were analyzed using MO Affinity Analysis software v2.3 (NanoTemper Technologies GmbH, Munich, Germany). The binding affinity was presented as normalized fluorescence (F_Norm_ [‰]) (71).

### Thermal stability characterization

Thermal stability of α-SplB1 was determined in PBS by nano-differential scanning fluorimetry (nanoDSF) using the NanoTemper Prometheus NT.48 (NanoTemper Technologies GmbH, Munich, Germany). It uses intrinsic protein fluorescence to monitor structural changes (folding/unfolding) while applying a defined temperature profile. The α-SplB1 samples were set to a concentration of 2 mg/mL and heated from 20 °C to 95 °C at Δ0.5 °C/min.

### Protein extraction from *S. aureus* culture supernatant

ECPs were extracted from *S. aureus* strains NCTC 8325–4 (21) and D29, a community-acquired USA300 derivative (22), both belonging to the clonal complex CC8. Overnight cultures of each of the two strains in TSB medium were used to inoculate the main cultures in TSB medium to an OD at 600 nm of 0.05. Main cultures were grown at 37°C with linear shaking (150 strokes/min) in a water bath (Grant Instruments, Royston, UK), and the culture supernatants were collected at early stationary phase after 7 h (OD at 600 nm ~9) (4°C, 10 min, 4,700 × *g*). ECPs were precipitated from 1 mL of culture supernatants using trichloroacetic acid at a final concentration of 15% (v/v) for 48 h at 4°C and collected by centrifugation (1 h, 4°C, 17,000 × *g*). The resulting protein pellets were washed and centrifuged (10 min, 4°C, 17,000 × *g*) at least four times using pre-cooled 70% (v/v) ethanol and finally rinsed using pre-cooled 100% ethanol. After air drying, the protein pellets were dissolved in 20 mM HEPES (pH 8) with 1% (w/v) sodium dodecyl sulfate (SDS). Protein concentrations were determined using a bicinchoninic acid assay (Micro BCA Protein-Assay-Kit, ThermoFisher Scientific, Waltham, USA) according to the manufacturer’s instructions against bovine serum albumin (10–160 µg/µL) as standard, using the best-fit regression line.

### Quantitative Western blot for SplB

For Western blot, 5 µg of ECPs of *S. aureus* strains NCTC 8325-4 and D29 as well as serially diluted tag-free SplB were denatured for 5 min at 95°C and separated by 1D-SDS PAGE on a 4%–12% Bis-Tris protein gel (NuPAGE, ThermoFisher Scientific, Waltham, USA) at 150 V for about 40 min. Protein transfer to a PVDF membrane was performed using the Bio-Rad Trans-Blot Turbo Transfer System according to the manufacturer’s instructions (Bio-Rad, Hercules, USA).

Protein detection was done using the LI-COR system (LI-COR Biosciences, Lincoln, USA), according to the manufacturer’s instructions with slight adaptations, described earlier (72). In brief, for total protein detection, we utilized the LI-COR Revert Total Protein Stain protocol. After destaining and blocking, an overnight incubation of the membrane at 4°C using the primary α-SplB1 antibody (300 ng/mL in *Intercept* T20 AB Diluent, LI-COR Biosciences, Lincoln, USA) was performed. After washing, the membrane was incubated for 1 h at RT using the IRDye 800CW goat-anti mouse IgG secondary antibody (1:10,000 in *Intercept* T20 AB Diluent containing 0.01% [v/v] SDS, LI-COR Biosciences, Lincoln, USA). After washing and drying for 5 min at 50°C, signals were detected at 800 nm using the LI-COR Odyssey CLx Scanner (LI-COR Biosciences, Lincoln, USA).

Using Image Studio version 5.2.5 (LI-COR Biosciences, Lincoln, USA), signal areas were calculated, and background intensities were obtained by the averaged background above and below the signal area. The amount of SplB in the supernatant of *S. aureus* strains NCTC 8325-4 and D29 was calculated using the signal intensities of the tag-free SplB standard (SplB standard curve, Fig. S4), using a polynomial trend line.

### Quantitative Western blot for C3

SplB (0.5 µM) was pre-incubated with αSplB1 mAb at a 1:1 or 1:2 molar ratio or with PBS as control. Afterwards, the enzymatic activity of SplB was assessed by analyzing the cleavage of the SplB substrate human C3 (0.165 µM) (Complement Technology Inc, Texas, USA) at 37°C for 6 h. The samples were separated by 1D-SDS-PAGE under reducing conditions and transferred to a PVDF membrane as described above. Membranes were incubated with polyclonal rabbit anti-human C3 (1:2000, PA5-21349, ThermoFisher Scientific, Waltham, USA), washed, and subsequently incubated with IRDye 680RD goat anti-rabbit IgG (1:10,000, LI-COR Biosciences, Lincoln, USA). Signals were detected and analyzed as described above.

### Substrate cleavage assay with recombinant SplB

A substrate-specific neutralization assay was conducted using the synthetic peptide substrate acetyl-L-valyl-L-glutamyl-L-isoleucyl-L-aspartic acid (Ac-VEID) that is covalently linked to α−4-methyl-coumaryl-7-amide (MCA) (Peptanova, Sandhausen, Germany), which harbors the cleavage site for SplB (73). Upon proteolytic cleavage, MCA is converted into the fluorescent 7-amino-4-methyl-cumarin (AMC), which can be quantified in a fluorescence reader. In the assay, 25 µM substrate was incubated with either 2.5 µM tag-free SplB alone or 2.5 µM SplB pretreated with different molar ratios (1:1, 1:2, 1:4, and 1:8) of α-SplB1 at 37°C for 1 h. 5 µM α-SplB1 mAb served as a negative control. The fluorescent AMC signal was continuously measured over 60 min on a TECAN Infinite M200 plate reader (Tecan Group Ltd., Maennedorf, Switzerland; excitation: 360 nm, emission: 460 nm). Starting fluorescence intensities for each assay condition were adjusted in order to obtain the same t0 values.

### Substrate cleavage assay with *S. aureus* supernatants

Three *splB*-negative *S. aureus* isolates (Xdlps 447 [CC45], SN2336 [CC22], SN2295 [CC59]) and three *splB*-positive isolates (Xdlps 319 [CC25], SN2242 [CC5], and SN2252 [CC8]) were cultured in TSB medium, and supernatants were harvested at early stationary phase (5.5 h, OD at 600 nm ~4.0). To enrich the secreted SplB and remove medium components, 50 mL of supernatants was concentrated 250-fold using two steps of size exclusion centrifugation with Amicon Ultra 30K/10K centrifugal filter units (Merck Millipore, Billerica, USA). Using serially diluted recombinant SplB as a standard, the concentration of native SplB in the supernatants was estimated to be <0.005 µM for the *splB*-negative strains and ~1.0 µM (range: 0.9–1.2) for the *splB*-positive strains. Supernatants were mixed with the α-SplB1 antibody (0.38, 0.75, 1.5, and 3.0 µM) and incubated for 1 h at RT. Controls were mixed with an equal volume of PBS. Afterwards, 80 µL of the Ac-VEID-MCA substrate (25 µM) were added, and the substrate cleavage was quantified over 240 min as described above.

### Mouse model of cremaster muscle vascular leakage

To assess the *in vivo* neutralizing capabilities of α-SplB1, we used a mouse cremaster muscle microvascular leakage model. The animal experiment complied with German animal protection laws and was approved by the local animal protection authority (Government of Oberbayern, Munich, Germany; AZ ROB-55.2Vet-2532.Vet_02-17-68).

The surgical preparation of the mouse cremaster muscle was performed as previously described (74). Briefly, male C57BL/6N wild-type mice, aged 6–8 weeks, were sedated with ketamine/xylazine (100 mg/kg ketamine and 10 mg/kg xylazine; i.p.). The left femoral artery was then cannulated in a retrograde manner using a polyethylene-10 catheter (inner diameter 0.28 mm, Clay Admas, New York, USA) to facilitate the administration of tag-free SplB with or without α-SplB1. Then, a ventral incision was made in the scrotum to expose the right cremaster muscle, which was placed over a transparent glass pedestal on a custom-made microscopy stage. The epididymis and testicle were detached from the cremaster muscle and placed in the abdominal cavity. Throughout the procedure, the muscle was continuously perfused with warm buffered saline at 37°C to maintain its metabolic and physiological activity. After intrascrotal stimulation with 10 µg tag-free SplB, leakage of 50 µL intravenously applied FITC-dextran (150 kDa, Sigma-Aldrich, St. Louis, USA) to the perivascular adipose tissue was analyzed in the mice. In a parallel experiment, mice received α-SplB1 (150 µg, corresponding to a 1:2.2 molar ratio of SplB: α-SplB1) 1 h prior to SplB administration to assess if α-SplB1 could neutralize the effect of SplB. The FITC-dextran signal was visualized using fluorescence microscopy (Zeiss, Oberkochen, Germany) (excitation: 488 nm; emission: 515 nm), and the images were captured with a CCD camera (Sensicam, PCO, Kelheim, Germany). Mean fluorescence intensity (MFI) values were measured in six randomly selected regions of interest (ROIs, 50 × 50 µm²) approximately 50 µm away from the observed postcapillary venule, utilizing ImageJ software (Maryland, USA). Data were presented as mean MFI ± SD of 3–8 animals per group.

### Statistical analyses

Statistical testing and data visualization were performed with GraphPad Prism (v8.0.1). An unpaired *t*-test was employed for comparison of two datasets (*P < 0.05).

## SUPPLEMENTAL MATERIAL

Supplemental material iai.00171-25-s0001.pdf Fig. S1 to S5; Table S1.

ASM does not own the copyrights to Supplemental Material that may be linked to, or accessed through, an article. The authors have granted ASM a non-exclusive, world-wide license to publish the Supplemental Material files. Please contact the corresponding author directly for reuse.
