# Supplementary material for: Monoclonal antibody neutralizes Staphylococcus aureus serine protease-like protein B (SplB)-induced pathology
Source: Infect Immun. 2025 Jun 13;93(7):e00171-25. doi: 10.1128/iai.00171-25 (PMC12234435; doi:10.1128/iai.00171-25)
Supplement: Supplemental material — Fig. S1 to S5; Table S1. [file iai.00171-25-s0001.pdf]

Supplementary Figure 1

>α-SplB1 _LC_DNA
TGACATTCTGATGACCCAGTCTCCAGCCACCCTGTCTGTGACTCCAGGAGATAGAGTCTCTCTTTCCTG
CAGGGCCAGTCAGAGTATTAGCGACTACTTACACTGGTATCAACAAAAATCACATGAGTCTCCAAGGC
TTCTCATCAAATATGCTTCCCAATCCATCTCTGGGATCCCCTCCAGGTTCAGTGGCAGTGGATCAGGGT
CAGATTTCACTCTCAGTATCAACAGTGTGGAACCTGAAGATGTTGGAGTGTATTACTGTCAAAATGGT
CACATCTTTCCTCCCACGTTCGGTGCTGGGACCAAGCTGGAGCTGAAACGGGCTGATGCTGCACCAA
CTGTATCCATCTTCCCACCATCCATGAGCAGTTAACATCTGGAGA

>α-SplB1 _LC_AA
DILMTQSPATLSVTPGDRVSLSCRASQSISDYLHWYQQKSHESPRLLIKYASQSISGIPSRFSGSGSGSDFTL
SINSVEPEDVGVYYCQNGHIFPPTFGAGTKLELKRADAAPTVSIFPPSMSS

>α-SplB1 _HC_DNA
TTTGGGAATTCGAGGTGCAGCTGCAGGAGTCTGGCCCTGGGATATTGCAGTCTTCCCAGACCCTCAG
TCTGACTTGTTCTTTCTCTGGGTTTTCACTGAACTCTGTTGGTATGGGTGTGACCTGGATTCGTCAGCC
TTCAGGAAAGGGTCTGGAGTGGCTGGCACACATTTACTGGGATGATGACAAGCTCTATAATTCATCCC
TGAAGAGCCGGCTCACAATCTCCAAGGATACCTCCAGAAACCAGGTTTTCCTCAATATCACCAGTGTG
GTCTCTGCAGATACTGCCACATACTACTGTGCTCGAAGAGTCTACGGTATTAGCTACGACTACTGGTAC
TTCGATGTCTGGGGCACAGGGACCGCGGTCACCGTCTCCTCAGCCAAAACGACACCCCCATCTGTCT
ATCCACTGGCCCCCTGGGTG

>α-SplB1 _HC_AA
WEFEVQLQESGPGILQSSQTLSLTCSFSGFSLNSVGMGVTWIRQPSGKGLEWLAHIYWDDDKLYNSSLK
SRLTISKDTSRNQVFLNITSVVSADTATYYCARRVYGISYDYWYFDVWGTGTAVTVSSAKTTPPSVYPLAP
WV

Figure S1. DNA and amino acid sequences of the Fab region of the heavy and light chains of the α-SplB1 mAb.

Supplementary Figure 2

A **α-SplB1 - light chain**

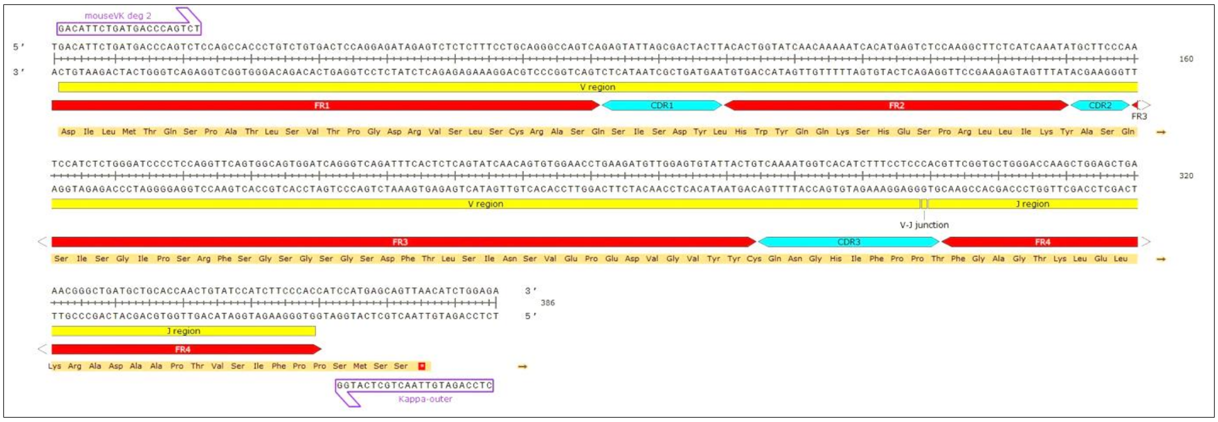

α-SplB1 - heavy chain

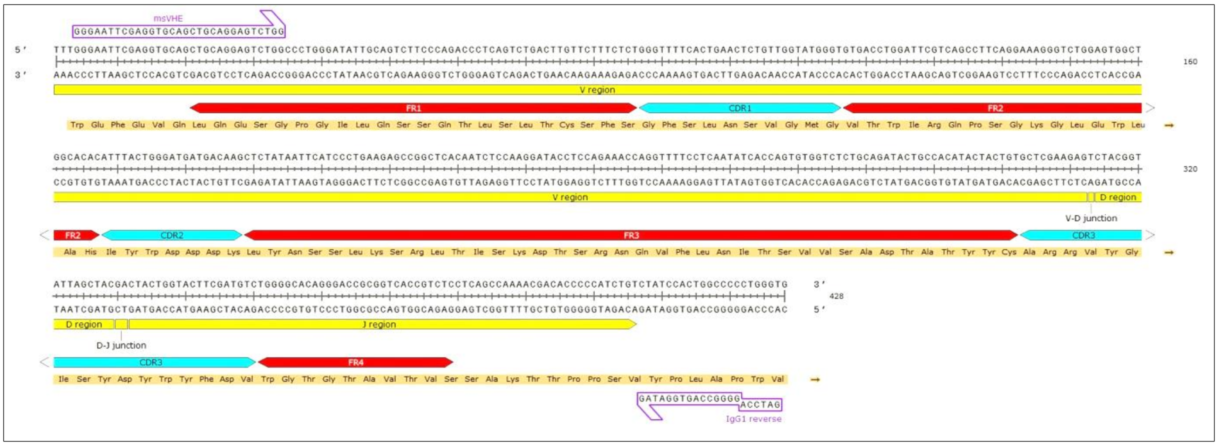

B

Name	Germline NCBI accession number	Region	From (bp)	To (bp)	Length (bp)	Matches	Mismatches	Gaps	Identity (%)	Silent Mutations	Non-silent mutations	Amino acid changes
α-SplB1 HC	NC_000078.6	FR1	21	86	66	63	3	0	95.5	2	6	2
		CDR1	87	116	30	26	4	0	86.7	0	4	2
		FR2	117	167	51	50	1	0	98	0	1	0
		CDR2	168	188	21	21	0	0	100	0	0	0
		FR3	189	302	114	107	7	0	93.9	2	5	4
		CDR3	303	312	10	10	0	0	100	0	0	0
		Total			292	277	15	0	94.9	4	16	8
α-SplB1 LC	NC_000072.6	FR1	79	78	75	3	0	0	96.2	2	1	0
		CDR1	80	97	18	18	0	0	100	0	0	0
		FR2	98	148	51	51	0	0	100	0	0	0
		CDR2	149	157	9	9	0	0	100	0	0	0
		FR3	158	265	108	108	0	0	100	0	1	0
		CDR3	266	288	23	22	1	0	95.7	0	0	1
		Total			287	283	4	0	98.6	2	2	1

Figure S2. Annotated variable regions of light chain (LC) and heavy chain (HC) of α-SplB1 and their sequence identity to murine germline sequences. (A) RNA was isolated from the hybridoma culture producing α-SplB1 mAb and reverse-transcribed into cDNA. The gene regions encoding the variable regions of LC and HC of α-SplB1 were amplified and sequenced. Ig-BLAST software was used to analyze the DNA sequences of α-SplB1, annotate variable/diversity/joining (VDJ) or VJ regions of HC/LC (yellow), framework regions (FRs; red), and complementarity-determining regions (CDRs; light blue). Translated amino acid sequences are shown below the DNA sequences. Primers used for amplification of variable regions of HC/LC are highlighted in purple. The image was generated using SnapGene Viewer (version 5.0.7, SnapGene software (www.snapgene.com)). (B) Comparison of the variable region sequence of LC and HC of α-SplB1 with murine germline sequences. Abbreviations: bp, base pair; HC, heavy chain; LC, light chain

Supplementary Figure 3

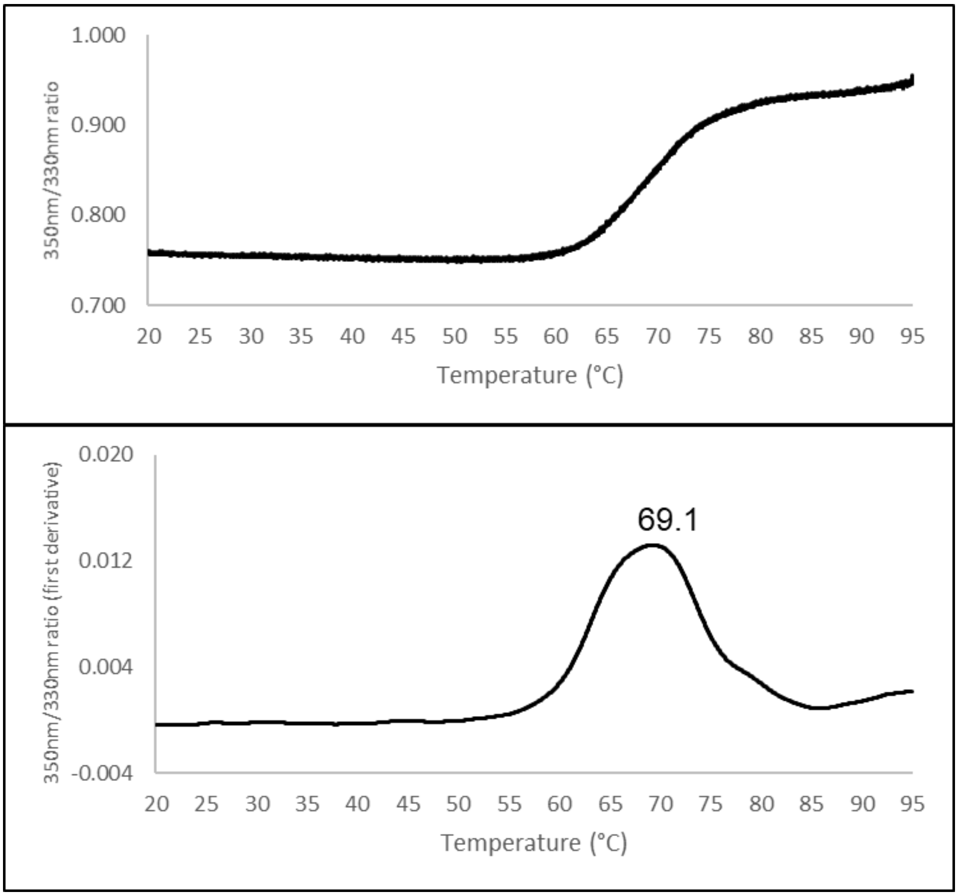

Figure S3: Thermal stability of α-SplB1 as determined by nano-differential scanning fluorimetry. Plots of F350/F330 fluorescence ratio (top) and its first derivative (bottom) versus temperature of 2 mg/mL α-SplB1 mAb in PBS. The antibody unfolded in a single melting transition with an onset of 58 $\pm$ 0.07 °C and melting temperature of 69.1 $\pm$ 0.05 °C (n = 3 measurements).

Supplementary Figure 4

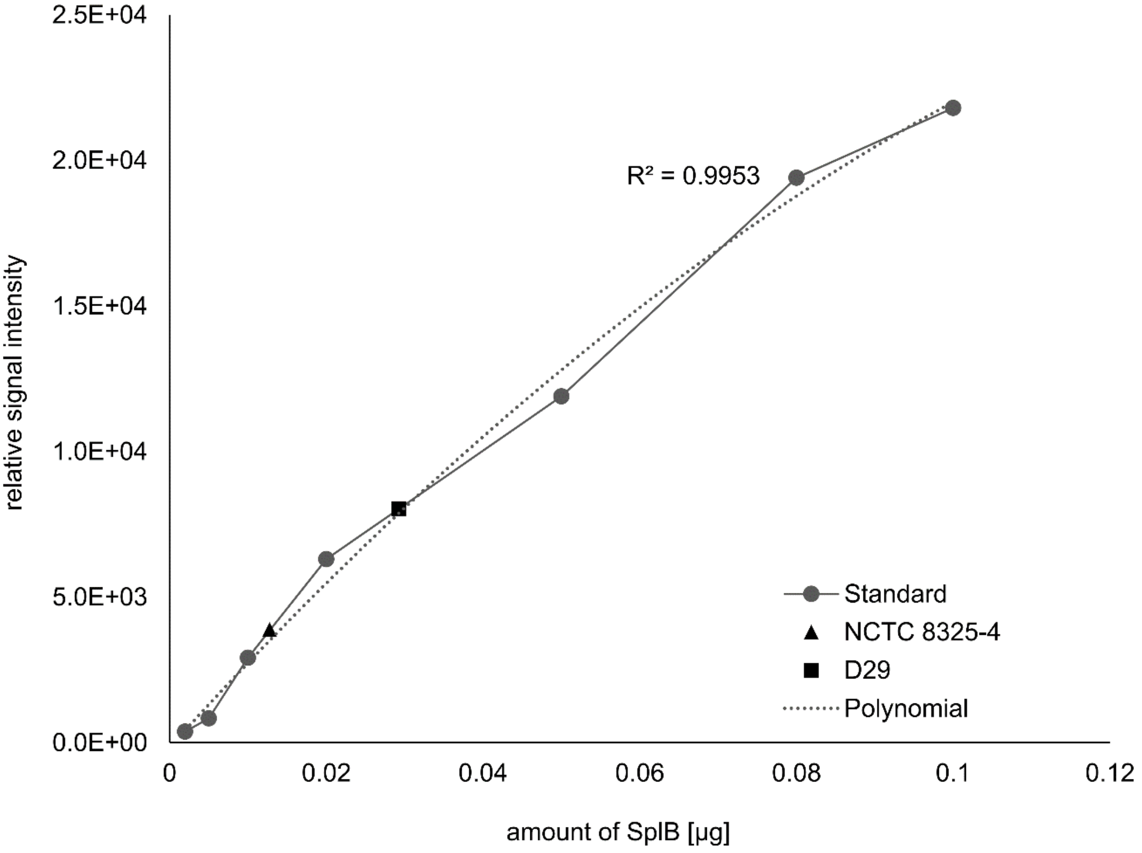

Figure S4: Standard curve of SplB quantification by Western blot. The absolute amount of SplB in the culture supernatant of the *S. aureus* isolates NCTC 8325-4 and D29 was determined by quantitative Western blot using an SplB standard curve based on polynomial regression. To determine the SplB concentration in the supernatants, proteins were precipitated from 1 mL of each supernatant, reconstituted in 40 µL buffer, and their concentration was determined using a BCA-assay. A total of 5 µg ECP from extract *S. aureus* NCTC 8325-4 (black triangle) and D29 (black square) contained 0.0139 µg and 0.0299 µg SplB, respectively. Accordingly, the SplB amount per 1 µg total ECP protein was calculated as 2.8 ng (NCTC 8325-4) and 6.0 ng (D29). The concentration of SplB in the staphylococcal culture supernatants during stationary growth phase in TSB was determined to be 527 ng/mL (NCTC 8325-4) and 541 ng/mL (D29).

Supplementary Figure 5

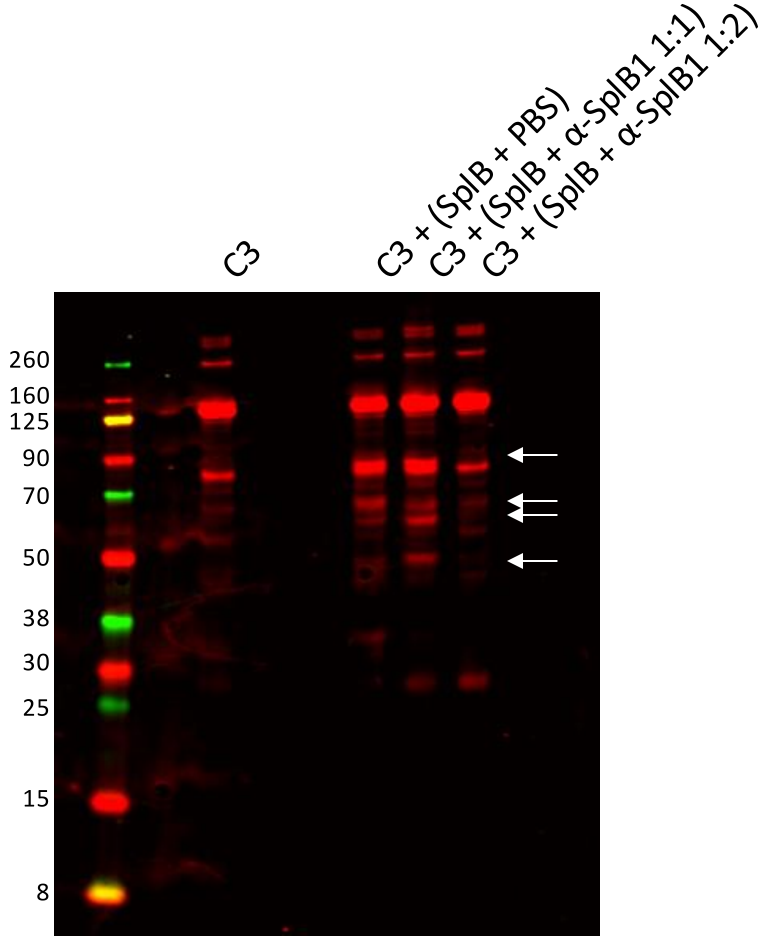

Figure S5: α-splB1 inhibits SplB-induced cleavage of human complement factor C3. SplB (0.5 μM) was preincubated with α-SplB1 at a 1:1 or 1:2 molar ratio for 1 hour, followed by incubation with human complement factor C3 (0.165 μM) for 6 h at 37 °C. Cleavage products (white arrows) were visualized by Western blot analysis using a polyclonal rabbit anti-human C3 (1:2,000) and IRDye 680RD goat anti-rabbit IgG (1:10,000). SplB-mediated C3 cleavage was neutralized by α-SplB1 at a 1:2 molar ratio (SplB:α-SplB1). MWs: C3 α chain: 115 kDa; C3 β chain: 75 kDa.

Supplementary Table 1

Table S1: List of primers used for the amplification of the variable regions of the heavy and light chains of α-SplB1.

Amplified region	Number	Name	Specifics	Sequence
Forward primer in the variable region				
**Heavy chain**	O839	VH deg	mouse and human universal VH degenerate primer, FR1	AGGTSMARCTGCAGSAGTCWGG
		msVHE	mouse and human universal VH degenerate primer, FR1	GGGAATTCGAGGTGCAGCTGCAGGAG TCTGG
**Light chain**	p647	mouse VK deg 2	universal primer for mouse Vκ amplification, FR1	GACATTCTGATGACCCAGTCT
	p648	mouse VK deg 1	universal primer for mouse Vλ amplification, FR1	CAGGCTGTTGTGACTCAGGAATCT
Reverse primer in the constant region				
**Heavy chain**	p348	IgM-inner-as	beginning of mouse constant region μ	GAAGACATTTGGGAAGGACTGACT
	p350	IgG1-inner-as	beginning of mouse constant region γ1	ATGGAGTTAGTTTGGGCAGCAGAT
	p354	IgG2b-inner-as	beginning of mouse constant region γ2b	AGGAACCAGTTGTATCTCCACACC
	p616	IgG2c-inner-as	beginning of mouse constant region γ2c	GAGCCAGTTGTACCTCCACACAC
	p614	IgG3-inner-as	beginning of mouse constant region γ3	AGGGACCAAGGGATAGACAGATG
	-	IgG1 reverse	beginning of mouse constant region γ1	GATCCAGGGGCCAGTGGATAG
	-	IgG2b reverse	beginning of mouse constant region γ2b	CACCCAGGGGCCAGTGGATAG
	-	IgG2a	beginning of mouse constant region γ2a	CACGCAGGGGCC AGTGGATAG
	-	IgG2c inner	beginning of mouse constant region γ2c	GCTCAGGGAAATAACCCTTGAC
	-	IgG3 inner	beginning of mouse constant region γ3	GCTCAGGGAAGTAGCCTTTGAC
**Light chain**	p355	Kappa-outer	beginning of mouse constant region κ	CTCCAGATGTTAACTGCTCATGG
	p357	mlC1-outer	beginning of mouse constant region λ1	ATCTACCTTCCAGTCCACTGTCAC
	p358	mlC23-outer	beginning of mouse constant region λ2, 3	ATTTGCCTTCCAGGCCACTGTCAC

Abbreviations: FR, framework region; VH, heavy chain variable domain; Vκ, variable domain of kappa light chain; Vλ, variable domain of lambda light chain; S: G or C; M: A or C; R: A or G; W: A or T.
